# *N*,*N*′-Dicyclo­hexyl­ethyl­enediammonium dichloride

**DOI:** 10.1107/S1600536809052222

**Published:** 2009-12-12

**Authors:** Bob-Dan Lechner, Kurt Merzweiler

**Affiliations:** a Institut für Chemie, Naturwissenschaftliche Fakulät II, Martin-Luther-Universität Halle-Wittenberg, Kurt-Mothes-Str. 2, 06120 Halle, Germany

## Abstract

In the title compound, C_14_H_30_N_2_^2+^·2Cl^−^, the *N*,*N*′-dicyclo­hexyl­ethyl­enediammonium cation posseses crystallographic 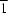 symmetry, and thus the compound crystallizes with two formula units per unit cell. In the crystal, the cations and anions are linked by N—H⋯Cl hydrogen bonds, giving a two-dimensional network with {6,3} topology.

## Related literature

For the crystal structures of cyclo­hexyl­ammonium derivatives, see Smith *et al.* (1994 ▶); Martell & Zaworotko (1991 ▶). For the crystal structure of an iridium complex with the *N*,*N*′-dicyclo­hexyl­ethyl­enediamine ligand, see: Greulich *et al.* (2002 ▶). For a review of hydrogen bonding, see Steiner (2002 ▶). *N*,*N*′-dicyclo­hexyl­ethyl­enediamine was prepared according to Denk *et al.* (2003 ▶). For the topology of {6,3} ring systems and three-dimensional polyhedra and networks, see: Wells & Sharpe (1963 ▶).
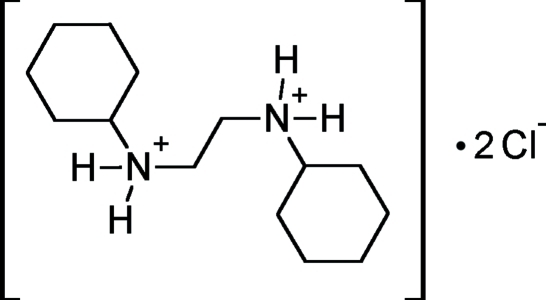


## Experimental

### 

#### Crystal data

C_14_H_30_N_2_^2+^·2Cl^−^
*M* *_r_* = 297.30
Monoclinic, 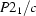

*a* = 11.551 (3) Å
*b* = 6.785 (2) Å
*c* = 10.8434 (17) Å
β = 91.892 (15)°
*V* = 849.3 (4) Å^3^
*Z* = 2
Mo *K*α radiation
μ = 0.37 mm^−1^
*T* = 293 K
0.65 × 0.28 × 0.12 mm

#### Data collection

Stoe STADI4 diffractometer
3349 measured reflections
1675 independent reflections
1430 reflections with *I* > 2σ(*I*)
*R*_int_ = 0.036
2 standard reflections every 120 min
intensity decay: none

#### Refinement

*R*[*F* ^2^ > 2σ(*F* ^2^)] = 0.035
*wR*(*F* ^2^) = 0.089
*S* = 1.10
1675 reflections
142 parameters
H atoms treated by a mixture of independent and constrained refinement
Δρ_max_ = 0.23 e Å^−3^
Δρ_min_ = −0.28 e Å^−3^

### 

Data collection: *STADI4* (Stoe & Cie, 1996 ▶); cell refinement: *STADI4*; data reduction: *X-RED* (Stoe & Cie, 1996 ▶); program(s) used to solve structure: *SHELXS97* (Sheldrick, 2008 ▶); program(s) used to refine structure: *SHELXL97* (Sheldrick, 2008 ▶); molecular graphics: *DIAMOND* (Brandenburg, 2009 ▶); software used to prepare material for publication: *SHELXL97* and *PLATON* (Spek, 2009 ▶).

## Supplementary Material

Crystal structure: contains datablocks I, global. DOI: 10.1107/S1600536809052222/si2221sup1.cif

Structure factors: contains datablocks I. DOI: 10.1107/S1600536809052222/si2221Isup2.hkl

Additional supplementary materials:  crystallographic information; 3D view; checkCIF report

## Figures and Tables

**Table 1:** Hydrogen-bond geometry (Å, °)

*D*—H⋯*A*	*D*—H	H⋯*A*	*D*⋯*A*	*D*—H⋯*A*
N—H4⋯Cl	0.91 (2)	2.20 (2)	3.1088 (16)	175.8 (18)
N—H3⋯Cl^i^	0.84 (2)	2.30 (2)	3.1250 (16)	168.8 (18)

Symmetry code: (i) 
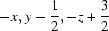
.

**Fig. 1.**
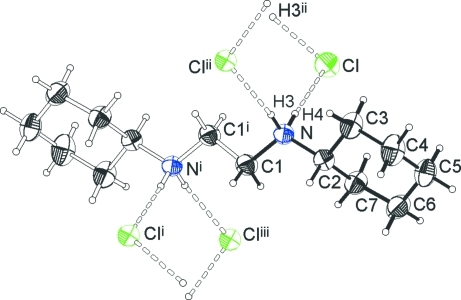
Molecular structure of the [CyNH2CH2CH2NH2Cy]2+ cation with surrounding Cl- anions. The asymmetric unit is shown by filled bonds. Thermal ellipsoids are drawn at the 50% probability level. Symmetry codes: (i) -x, -y, -z; (ii) -x, -1/2 + y, 1.5 - z; (iii) x, 1.5 - y, -1/2 + z

**Fig. 2.**
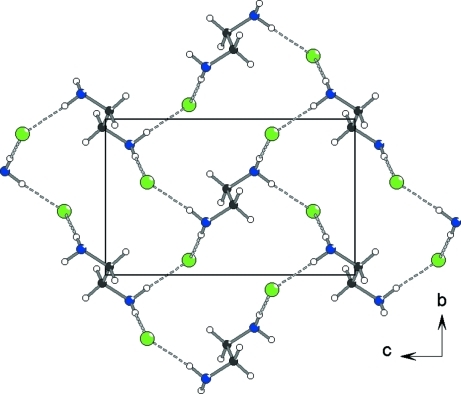
Part of the layer structure formed by hydrogen bonded [CyNH2CH2CH2NH2Cy]2+ cations and Cl- anions. Cyclohexyl groups are omitted for clarity.

## supplementary crystallographic information

### Comment

Compared to other *N*,*N*'-disubstituted ethylenediamine compounds RNH-CH_2_CH_2_—NHR (*R* = Me, Ph, *etc*.) *N*,*N*'-Dicyclohexylethylenediamine derivatives have been studied only rarely by X-ray diffraction. One of the few examples is the Iridium complex [Cp*(CyNHCH_2_CH_2_NHCy)HIr][H_3_BCN] (Greulich *et al.*, 2002).

The crystal structure of the title compound (Fig. 1) consists of [CyNH_2_CH_2_CH_2_NH_2_Cy]^2+^ cations and Cl^- ^anions. The [CyNH_2_CH_2_CH_2_NH_2_Cy]^2+ ^cations exhibit crystallographic 1 symmetry and thus an exactly staggered conformation with a N—C—C—N torsion angle of 180 ° is observed. The N atoms display a distorted tetrahedral coordination with H—N—C angles of 108.6 to 111.2 and a C—N—C angle of 114.50 (1)°. The cyclohexyl groups adopt a chair conformation (Fig. 2) as it was observed in [Cp*(CyNHCH_2_CH_2_NHCy)HIr][H_3_BCN] (Greulich *et al.*, 2002). Both hydrogen atoms of the NH_2_ groups are involved in hydrogen bridges to neighbouring Cl^-^ anions. The NH···Cl distances of 2.20 (2) and 2.30 (2) Å are comparable to those found in other cyclohexylammonium derivatives like [CyNH_3_]Cl (2.187–2.35.4 Å) (Smith *et al.*, 1994) and [CyNH_3_]_2_(AlCl_4_)Cl (2.305–2.478 Å) (Martell & Zaworotko, 1991) respectively. The N—H···Cl angles of 169 (2)° and 176 (2)° are in the expected range for hydrogen bridges of moderate strength (Steiner, 2002).

On balance each [CyNH_2_CH_2_CH_2_NH_2_Cy]^2+^ cation forms four N—H···Cl bridges to neighbouring Cl^-^ anions and each Cl^-^ anion acts as H-acceptor for two NH hydrogen atoms. As a result of the hydrogen bonding between [CyNH_2_CH_2_CH_2_NH_2_Cy]^2+^ cations and Cl^-^ anions a two-dimensional layer structure is formed. The layers consist of puckered C_4_H_8_N_6_Cl_4_ rings that are interconnected to give a honeycomb arrangement with {6,3} net topology (Wells & Sharpe, 1963).

### Experimental

An excess of hydrochloric acid was added dropwise to a solution of *N*,*N*'-Dicyclohexylethylenediamine monohydrate (1.11 g, 5 mmol) prepared by standard techniques (Denk *et al.*, 2003) in a ethanol/water mixture(10:1, 20 ml) and stirred for 6 h at 140 °C in an autoclave. The mixture was slowly cooled to ambient temperature and colourless plate-like crystals were obtained. Spectroscopic data: ^1^H NMR (D_2_O, 500 MHz, 298 K, p.p.m.): δ 1.07–1.99 (m, 20 H, CH_2, _Cy), 3.09 (m, 2H, CH), 3.32 (s, 4H, CH_2_); ^13^C NMR (D_2_O, 125 MHz, 298 K, p.p.m.): δ 23.7 (s, CH_2_, Cy: C4, C6), 24.3 (s, CH_2_, Cy: C5), 26.7 (s, CH_2_, Cy: C3, C7), 40.0 (s, CH_2_), 57.934 (s, CH, Cy).

### Crystal data

**Table:**

C_14_H_30_N_2_^2+^·2Cl^−^	*F*(000) = 324
*M**_r_* = 297.30	*D*_x_ = 1.163 Mg m^−^^3^
Monoclinic, *P*2_1_/*c*	Mo *K*α radiation, λ = 0.71073 Å
Hall symbol: -P 2ybc	Cell parameters from 22 reflections
*a* = 11.551 (3) Å	θ = 10.2–14.6°
*b* = 6.785 (2) Å	µ = 0.37 mm^−^^1^
*c* = 10.8434 (17) Å	*T* = 293 K
β = 91.892 (15)°	Plate, colourless
*V* = 849.3 (4) Å^3^	0.65 × 0.28 × 0.12 mm
*Z* = 2	

### Data collection

**Table:**

Stoe STADI4 diffractometer	*R*_int_ = 0.036
Radiation source: fine-focus sealed tube	θ_max_ = 26.1°, θ_min_ = 1.8°
graphite	*h* = −14→14
2θ/ω scans	*k* = −8→0
3349 measured reflections	*l* = −13→13
1675 independent reflections	2 standard reflections every 120 min
1430 reflections with *I* > 2σ(*I*)	intensity decay: none

### Refinement

**Table:**

Refinement on *F*^2^	Primary atom site location: structure-invariant direct methods
Least-squares matrix: full	Secondary atom site location: difference Fourier map
*R*[*F*^2^ > 2σ(*F*^2^)] = 0.035	Hydrogen site location: inferred from neighbouring sites
*wR*(*F*^2^) = 0.089	H atoms treated by a mixture of independent and constrained refinement
*S* = 1.10	*w* = 1/[σ^2^(*F*_o_^2^) + (0.0304*P*)^2^ + 0.223*P*] where *P* = (*F*_o_^2^ + 2*F*_c_^2^)/3
1675 reflections	(Δ/σ)_max_ < 0.001
142 parameters	Δρ_max_ = 0.23 e Å^−^^3^
0 restraints	Δρ_min_ = −0.28 e Å^−^^3^

### Special details

**Table:**

Geometry. All e.s.d.'s (except the e.s.d. in the dihedral angle between two l.s. planes) are estimated using the full covariance matrix. The cell e.s.d.'s are taken into account individually in the estimation of e.s.d.'s in distances, angles and torsion angles; correlations between e.s.d.'s in cell parameters are only used when they are defined by crystal symmetry. An approximate (isotropic) treatment of cell e.s.d.'s is used for estimating e.s.d.'s involving l.s. planes.
Refinement. Refinement of *F*^2^ against ALL reflections. The weighted *R*-factor *wR* and goodness of fit *S* are based on *F*^2^, conventional *R*-factors *R* are based on *F*, with *F* set to zero for negative *F*^2^. The threshold expression of *F*^2^ > σ(*F*^2^) is used only for calculating *R*-factors(gt) *etc*. and is not relevant to the choice of reflections for refinement. *R*-factors based on *F*^2^ are statistically about twice as large as those based on *F*, and *R*- factors based on ALL data will be even larger.

### Fractional atomic coordinates and isotropic or equivalent isotropic displacement parameters (Å^2^)

**Table:**

	*x*	*y*	*z*	*U*_iso_*/*U*_eq_	
C1	0.05608 (15)	0.4477 (3)	0.48641 (16)	0.0404 (4)	
H2	0.1138 (18)	0.537 (3)	0.4631 (19)	0.054 (6)*	
H1	0.0449 (18)	0.353 (3)	0.419 (2)	0.059 (6)*	
C2	0.21086 (13)	0.2307 (2)	0.58171 (14)	0.0342 (3)	
H5	0.2039 (14)	0.163 (3)	0.5053 (16)	0.038 (4)*	
C3	0.22533 (16)	0.0777 (3)	0.6834 (2)	0.0478 (5)	
H6	0.2248 (18)	0.147 (3)	0.763 (2)	0.059 (6)*	
H7	0.1619 (18)	−0.009 (4)	0.6755 (18)	0.058 (6)*	
C4	0.33978 (18)	−0.0313 (3)	0.6726 (3)	0.0574 (5)	
H8	0.3397 (19)	−0.095 (3)	0.596 (2)	0.063 (7)*	
H9	0.346 (2)	−0.123 (4)	0.738 (2)	0.069 (7)*	
C5	0.44122 (17)	0.1104 (3)	0.6751 (2)	0.0513 (5)	
H10	0.4456 (18)	0.173 (3)	0.757 (2)	0.064 (6)*	
H11	0.516 (2)	0.045 (3)	0.668 (2)	0.068 (6)*	
C6	0.42621 (16)	0.2660 (3)	0.5755 (2)	0.0522 (5)	
H12	0.4254 (18)	0.198 (3)	0.497 (2)	0.064 (6)*	
H13	0.492 (2)	0.354 (4)	0.581 (2)	0.075 (7)*	
C7	0.31115 (15)	0.3757 (3)	0.58388 (18)	0.0419 (4)	
H14	0.3073 (17)	0.447 (3)	0.6637 (19)	0.054 (6)*	
H15	0.3005 (17)	0.466 (3)	0.5188 (18)	0.053 (6)*	
N	0.09763 (12)	0.3358 (2)	0.59677 (13)	0.0335 (3)	
H4	0.1015 (17)	0.416 (3)	0.6647 (19)	0.052 (6)*	
H3	0.0454 (17)	0.257 (3)	0.6169 (17)	0.045 (5)*	
Cl	0.12073 (4)	0.59342 (7)	0.83342 (4)	0.04938 (17)	

### Atomic displacement parameters (Å^2^)

**Table:**

	*U*^11^	*U*^22^	*U*^33^	*U*^12^	*U*^13^	*U*^23^
C1	0.0421 (9)	0.0418 (9)	0.0375 (8)	0.0074 (8)	0.0039 (7)	0.0097 (7)
C2	0.0376 (8)	0.0297 (8)	0.0351 (8)	0.0025 (6)	−0.0004 (6)	−0.0027 (6)
C3	0.0413 (10)	0.0349 (9)	0.0670 (13)	−0.0006 (8)	−0.0007 (8)	0.0165 (9)
C4	0.0521 (11)	0.0354 (9)	0.0838 (16)	0.0089 (9)	−0.0074 (10)	0.0073 (11)
C5	0.0405 (10)	0.0492 (11)	0.0638 (12)	0.0098 (8)	−0.0038 (8)	0.0007 (9)
C6	0.0391 (9)	0.0583 (12)	0.0596 (12)	0.0033 (9)	0.0088 (8)	0.0046 (10)
C7	0.0383 (9)	0.0361 (9)	0.0514 (10)	−0.0011 (7)	0.0039 (7)	0.0081 (8)
N	0.0338 (7)	0.0314 (7)	0.0352 (7)	−0.0018 (6)	0.0011 (5)	0.0043 (6)
Cl	0.0569 (3)	0.0446 (3)	0.0467 (3)	0.0160 (2)	0.00215 (19)	−0.00631 (19)

### Geometric parameters (Å, °)

**Table:**

C1—N	1.484 (2)	C4—H8	0.94 (2)
C1—C1^i^	1.514 (3)	C4—H9	0.95 (2)
C1—H2	0.94 (2)	C5—C6	1.516 (3)
C1—H1	0.98 (2)	C5—H10	0.98 (2)
C2—N	1.503 (2)	C5—H11	0.98 (2)
C2—C7	1.519 (2)	C6—C7	1.529 (2)
C2—C3	1.519 (2)	C6—H12	0.97 (2)
C2—H5	0.948 (18)	C6—H13	0.97 (3)
C3—C4	1.523 (3)	C7—H14	0.99 (2)
C3—H6	0.98 (2)	C7—H15	0.94 (2)
C3—H7	0.94 (2)	N—H4	0.91 (2)
C4—C5	1.515 (3)	N—H3	0.84 (2)
			
N—C1—C1^i^	109.80 (17)	C4—C5—C6	111.01 (17)
N—C1—H2	109.4 (13)	C4—C5—H10	108.0 (13)
C1^i^—C1—H2	111.7 (12)	C6—C5—H10	109.9 (13)
N—C1—H1	107.3 (13)	C4—C5—H11	113.4 (14)
C1^i^—C1—H1	111.1 (12)	C6—C5—H11	109.8 (13)
H2—C1—H1	107.4 (17)	H10—C5—H11	104.5 (18)
N—C2—C7	110.89 (13)	C5—C6—C7	112.07 (16)
N—C2—C3	108.69 (13)	C5—C6—H12	107.2 (13)
C7—C2—C3	111.44 (15)	C7—C6—H12	107.4 (13)
N—C2—H5	106.0 (10)	C5—C6—H13	108.4 (14)
C7—C2—H5	111.7 (10)	C7—C6—H13	112.4 (14)
C3—C2—H5	107.9 (11)	H12—C6—H13	109.2 (18)
C2—C3—C4	110.54 (17)	C2—C7—C6	110.34 (15)
C2—C3—H6	108.0 (12)	C2—C7—H14	105.8 (12)
C4—C3—H6	109.2 (12)	C6—C7—H14	110.7 (12)
C2—C3—H7	107.1 (13)	C2—C7—H15	109.3 (12)
C4—C3—H7	111.5 (13)	C6—C7—H15	111.5 (12)
H6—C3—H7	110.5 (17)	H14—C7—H15	109.1 (17)
C5—C4—C3	111.30 (17)	C1—N—C2	114.50 (13)
C5—C4—H8	106.8 (14)	C1—N—H4	110.6 (12)
C3—C4—H8	108.5 (14)	C2—N—H4	110.5 (13)
C5—C4—H9	111.3 (14)	C1—N—H3	108.6 (13)
C3—C4—H9	107.9 (14)	C2—N—H3	111.1 (13)
H8—C4—H9	111 (2)	H4—N—H3	100.6 (17)
			
N—C2—C3—C4	−179.40 (16)	C3—C2—C7—C6	55.7 (2)
C7—C2—C3—C4	−56.9 (2)	C5—C6—C7—C2	−54.8 (2)
C2—C3—C4—C5	56.5 (3)	C1^i^—C1—N—C2	−178.21 (18)
C3—C4—C5—C6	−55.5 (3)	C7—C2—N—C1	73.57 (19)
C4—C5—C6—C7	54.9 (2)	C3—C2—N—C1	−163.61 (15)
N—C2—C7—C6	176.95 (14)		

Symmetry codes: (i) −*x*, −*y*+1, −*z*+1.

### Hydrogen-bond geometry (Å, °)

**Table:**

*D*—H···*A*	*D*—H	H···*A*	*D*···*A*	*D*—H···*A*
N—H4···Cl	0.91 (2)	2.20 (2)	3.1088 (16)	175.8 (18)
N—H3···Cl^ii^	0.84 (2)	2.30 (2)	3.1250 (16)	168.8 (18)

Symmetry codes: (ii) −*x*, *y*−1/2, −*z*+3/2.
